# Synaptic inputs are tuned to match intrinsic properties to maintain phase in oscillatory neural networks

**DOI:** 10.1186/1471-2202-16-S1-P173

**Published:** 2015-12-18

**Authors:** Haroon Anwar, Jordan Storms, Farzan Nadim

**Affiliations:** 1 Federated Department of Biological Sciences, New Jersey Institute of Technology, Newark, NJ 07102, USA; 2 Rutgers University-Newark, Newark, NJ 07102, USA

## 

Rhythmic motor activity requires accurate temporal coordination of neurons controlling the muscles to produce a stable output pattern. Such coordinated activity manifests itself as the maintenance of the relative activity phases of the central pattern generating (CPG) neurons producing the rhythmic motor activity, in spite of a large range of network frequencies [1,2]. In many systems, this phase maintenance occurs even when the behavior is compared across different animals [1,2]. Surprisingly though, both the synaptic strengths [2] and voltage-gated ionic conductances [3] vary extensively across animals, leading to the question of how stability at the network level can arise despite such variability in the neuronal and synaptic components. We examine the hypothesis that phase maintenance in oscillatory networks involves a precise matching of the synaptic inputs to the neurons with the intrinsic properties of the neuron using the pyloric CPG network in stomatogastic nervous system (STNS) of crustaceans.

We used the biophysical model description of the pyloric follower LP bursting neuron [4] and a multi objective genetic algorithm to find multiple "optimal" models with varying levels of maximal conductance, each of which produced the appropriate bursting features (within the measured biological range) in response to the same periodic synaptic input. Additionally, all 24 optimal models exhibited tonic firing in the absence of synaptic input, as observed in biological LP neurons when synapses are blocked. We then removed the synaptic input to the LP neuron models and simulated dynamic clamp experiments by injecting 34 different synaptic conductance waveforms (which were recorded experimentally during ongoing rhythm) into the LP models, which, in most cases, switched the LP models from tonic firing to bursting. These simulations were repeated with the waveforms scaled to 500, 750, 1000, 1250, 1500, 1750 and 2000 ms, thus driving the LP neuron at these periods.

Our results show that when different LP models are injected with a unique synaptic waveform of fixed maximal amplitude the output varies greatly. Similarly, when a unique LP model is injected with different synaptic waveforms of fixed maximal amplitude the output also varies greatly. This indicates that not only the maximal amplitude of conductance but the other features of synaptic waveforms also play an important role in shaping the activity of LP neurons. Consequently, a distinct synaptic waveform would be required for each of the different LP model neurons to produce the same output features in LP neurons. Using this approach, we aim to explore what features of synaptic conductance are important determinant of changes in output features and how those synaptic properties match with different ionic conductances to maintain stereotypical output of pyloric network across animals. We further aim to confirm these results using dynamic clamp experiments in biological LP neurons with different synaptic waveforms. Our results also show that when the cycle period of each synaptic input is varied and applied to individual LP models, the phase of LP model neuron advances as expected. We aim to repeat all simulations with different maximal conductance values of synaptic waveforms. This will reveal the changes in synaptic strength required for the phase maintenance, when the cycle period is varied.
